# PREPs: An Open-Source Software for High-Throughput Field Plant Phenotyping

**DOI:** 10.34133/plantphenomics.0221

**Published:** 2024-08-09

**Authors:** Atsushi Itoh, Stephen N. Njane, Masayuki Hirafuji, Wei Guo

**Affiliations:** ^1^ Hokkaido Agricultural Research Center, National Agriculture and Food Research Organization, 9-4 Shinseiminami, Memurocho, Kasaigun, Hokkaido 082-0081, Japan.; ^2^ Graduate School of Agricultural and Life Sciences, The University of Tokyo, 1 Chome-1-1 Midoricho, Nishitokyo, Tokyo 188-0002, Japan.

## Abstract

An open-source software for field-based plant phenotyping, Precision Plots Analyzer (PREPs), was developed using Window.NET. The software runs on 64-bit Windows computers. This software allows the extraction of phenotypic traits on a per-microplot basis from orthomosaic and digital surface model (DSM) images generated by Structure-from-Motion/Multi-View-Stereo (SfM-MVS) tools. Moreover, there is no need to acquire skills in geographical information system (GIS) or programming languages for image analysis. Three use cases illustrated the software's functionality. The first involved monitoring the growth of sugar beet varieties in an experimental field using an unmanned aerial vehicle (UAV), where differences among varieties were detected through estimates of crop height, coverage, and volume index. Second, mixed varieties of potato crops were estimated using a UAV and varietal differences were observed from the estimated phenotypic traits. A strong correlation was observed between the manually measured crop height and UAV-estimated crop height. Finally, using a multicamera array attached to a tractor, the height, coverage, and volume index of the 3 potato varieties were precisely estimated. PREPs software is poised to be a useful tool that allows anyone without prior knowledge of programming to extract crop traits for phenotyping.

## Introduction

Precision agriculture has transformed farming through utilization of information technology (IT) for monitoring the crop growth condition. Similarly in breeding plots, the utilization of high-throughput phenotyping technologies has enabled the rapid and precise monitoring of the phenotype’s growth conditions. This has enabled not only the rapid breeding of new varieties over large scales but also the quantification of phenotypic traits and comparison of their growth properties under varying environmental conditions. For enhancement of crop breeding with high-throughput phenotyping, it is imperative to determine with high precision the parameters of crop height, volume, and coverage throughout crop growth. Recently, the utilization of remote sensing techniques such as unmanned aerial vehicle (UAV) has had viable applications owing to their low cost and quick return. However, the processing of the data obtained is marred by complicated software and rigorous, tedious programming languages that require specific computer environment settings and language-specific knowledge. Generating phenotypic traits using UAV involves several steps. First, the images obtained from the field are aligned using the Structure-from-Motion/Multi-View-Stereo (SfM-MVS) process, which estimates 3 dimensional parameters from the images. Second, densified point clouds and orthomosaic and DSM images are generated using SfM-MVS tools. Finally, to estimate crop height, volume, and coverage, several software packages such as Python-based Easy PCC [1] or QGIS (QGIS Development Team) are utilized. Using this software to generate phenotypic traits requires not only specific computer configurations but also a substantial investment of time to learn the programming languages. This requirement can be a barrier, particularly for breeders and agronomists who aim to generate information about phenotypic traits efficiently.

Thus far, several phenotyping platforms have been developed, particularly for monitoring the growth of crops from cellular levels to higher crops. This is because understanding the genotype and phenotype properties is important in improving crop traits and understanding the crop growth conditions from micro to macro based on different environmental conditions. CellProfiler, an open-source software used to analyze cell images, was developed and utilized to count cells, determine cell cycle distribution, and determine organelle numbers and sizes [2]. An affordable phenotyping system for capturing images, processing, and extracting root trait features has been developed [3]. An open-source and free breeding platform consisting of both mobile and web client interfaces has been developed, which enables users to collect, manage, and utilize breeding data for tomato, cucumber, and maize crops [4]. Arvidsson et al. [5] developed an automatic platform that incorporated annotation and automated modeling to estimate plant growth parameters such as leaf count to monitor the growth of *Arabidopsis thaliana* in growth cabinets in a controlled environment. However, this system could only be used indoors and was limited in that only strong plants could be detected without bias. Recently, efforts have been made to avail these phenotyping tools and data [6] for improving phenotyping methods and thus accelerating the development of disease-resistant and high-yielding breeds. Lobet et al. [7] developed a web repository to enable people to access the available software for phenotyping. It was found that cross-platform software has now been developed, enabling users to utilize it without the barriers to the operating system. A standalone software platform for analyzing hyperspectral data was developed and tested to generate the phenotypic traits of oilseed rape [8] from which drought stress can be determined early with high accuracy. An open source platform that could be implemented as a plugin in ImageJ was developed and utilized to determine the height, width, and shooting area of crops [9], and it can only be used for indoor phenotyping platforms. A deep segmentation phenotyping platform developed to generate training sets of data can be used to label objects, including defining tissue object boundaries, and thus train models for segmentation [10]. Similarly, PlantCV v2 open-source software enables individual plants from images with many plants to be analyzed, and leaf numbers can also be estimated [11]. However, currently available systems not only require lighting conditions but also are limited to indoor phenotyping facilities. The development of an open-source platform for analyzing open-field phenotyping systems is imperative.

In large open-field breeding plots, it is necessary to determine the properties of the phenotypes grown in microplots. Thus far, to determine such properties, manual extraction of the boundaries of these plots is performed using software such as QGIS, which requires not only an extensive period to learn how to utilize but also an overspecification because they are normally used for determining the boundaries of large areas such as countries. Tresch et al. [12] developed a program that enables segmentation and microplot extraction. However, to utilize this software, it is necessary that the field is homogeneous and that the crop rows do not touch each other, making it difficult to use, especially in advanced growth stages. Furthermore, phenotypic traits, such as height or coverage, could not be extracted using the software. User-friendly software for processing UAV and ground vehicle-based images can extract plot-level spectral data from soybean fields [13]. While a high correlation was obtained between the UAV-estimated and ground vehicle-estimated NDVI (normalized difference vegetation index) values, other plant phenotypic properties such as crop height or volume were not estimated.

In this study, we developed a graphical user interface (GUI)-based user-friendly image-processing software that enables the automatic extraction of phenotypic traits. The Precision Plots Analyzer (PREPs) software, developed within the Windows Form application of Microsoft’s .NET Framework, is designed to enable users to process their data without requiring programming skills. PREPs allow users to easily generate plot boundaries (popularly known as Shapefiles), automatically number them, adjust the plot number based on user requirements, and finally export the plots as Shapefiles for further processing of phenotypic traits. Normally, to determine the height of crops, the difference between the bare soil surface and the ground surface with the crops is utilized to estimate the crop height. However, some crops, such as potatoes, which are normally grown on ridges, lead to overestimation because the ridges are higher than the ground surface. PREPs allow users to select and utilize the top of the ridge as a reference plane, thereby ensuring the correct ground surface. Using an algorithm that calculates the smallest difference between each point on the ground, minute unevenness of the ground can be ignored. Using PREPs, the color pixels of crops and soil can be easily separated into different classes using a mouse, thus enabling the estimation of crop coverage. Finally, during the analysis, to prevent bias, selection can be made on whether to generate data from the whole plot or exclude some parts of the plot. Three use cases are reported to demonstrate the software. First, the growth of sugar beet varieties in an experimental field was monitored using a UAV, and the phenotypic traits, height, coverage, and volume index were estimated. Second, mixed European and Japanese potato varieties were monitored, and their respective phenotypic traits were extracted. A comparison was made between the ground truth and UAV-estimated crop heights to deduce the accuracy of the estimation. Third, as a proof of concept for the utilization of PREPs to process data collected from proximity sensors, a multicamera system attached to a tractor was utilized to monitor the growth of 3 Japanese potato varieties in an experimental field. The height, coverage, and volume index of potatoes were monitored with growth time, and a comparison was made with the ground-truth data to determine accuracy.

## System Functions

### Overview

**Fig. 1. Fig. 2.**
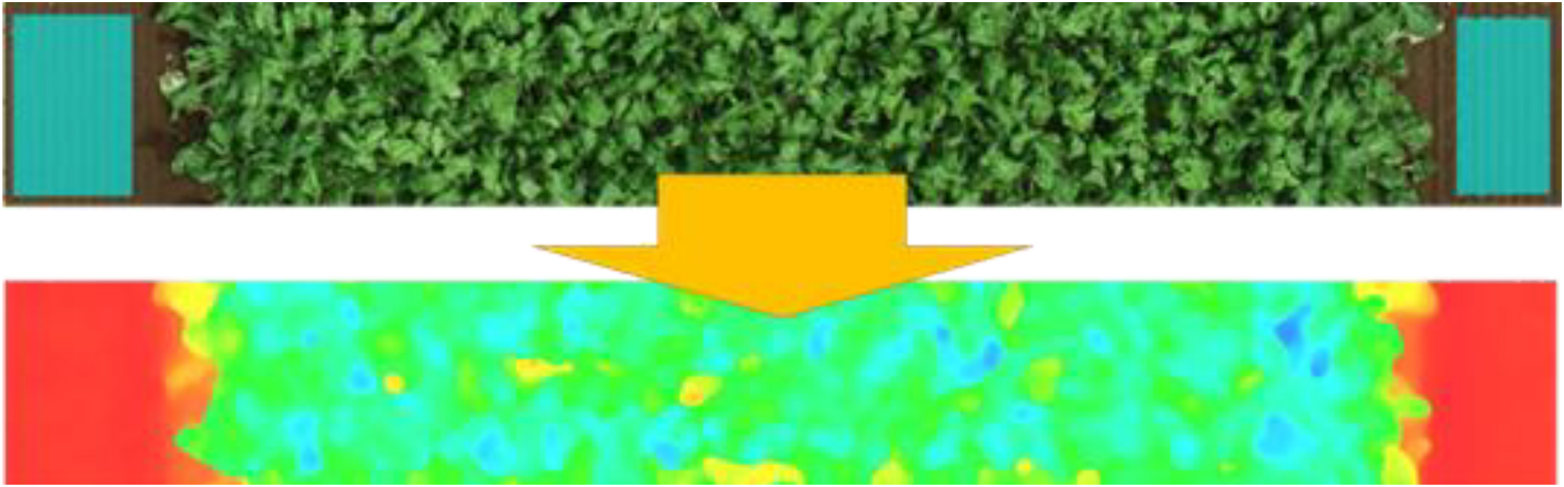
Base plane estimation using the side of the plot where the ground is visible (on the right sides) and hence utilized to calculate the base plane of the bare ground soil (now represented by red color)

PREPs is an open-source software designed for comprehensive analysis of field images. It was developed using Microsoft software. NET Framework (C#). This Windows Forms application is specifically designed to operate on 64-bit Windows OS platforms. The system is primarily designed for use in crop-breeding research fields, providing the function of dividing orthomosaic images into microplots and analyzing the images for each of these plots. A screenshot of a sample project in PREPs is shown in Fig. 1. The crop height, coverage, and volume index can be estimated from a pair of GeoTIFFs: orthomosaic RGB and digital surface model (DSM), generated by SfM-MVS using commercially available software such as Agisoft Metashape or Pix4D, as well as open-source alternatives like OpenDroneMap. Multiple orthomosaic RGB and DSM images from different capture dates can be registered and treated as time-series data. To ensure precise geospatial alignment of each image, ground control points (GCPs) were utilized during the capture and SfM processes. This ensured the accurate retention of spatial information within the images. The analysis results of this software can be accessed through lists and charts within the software interface and can also be exported in CSV format.

**Figure:**
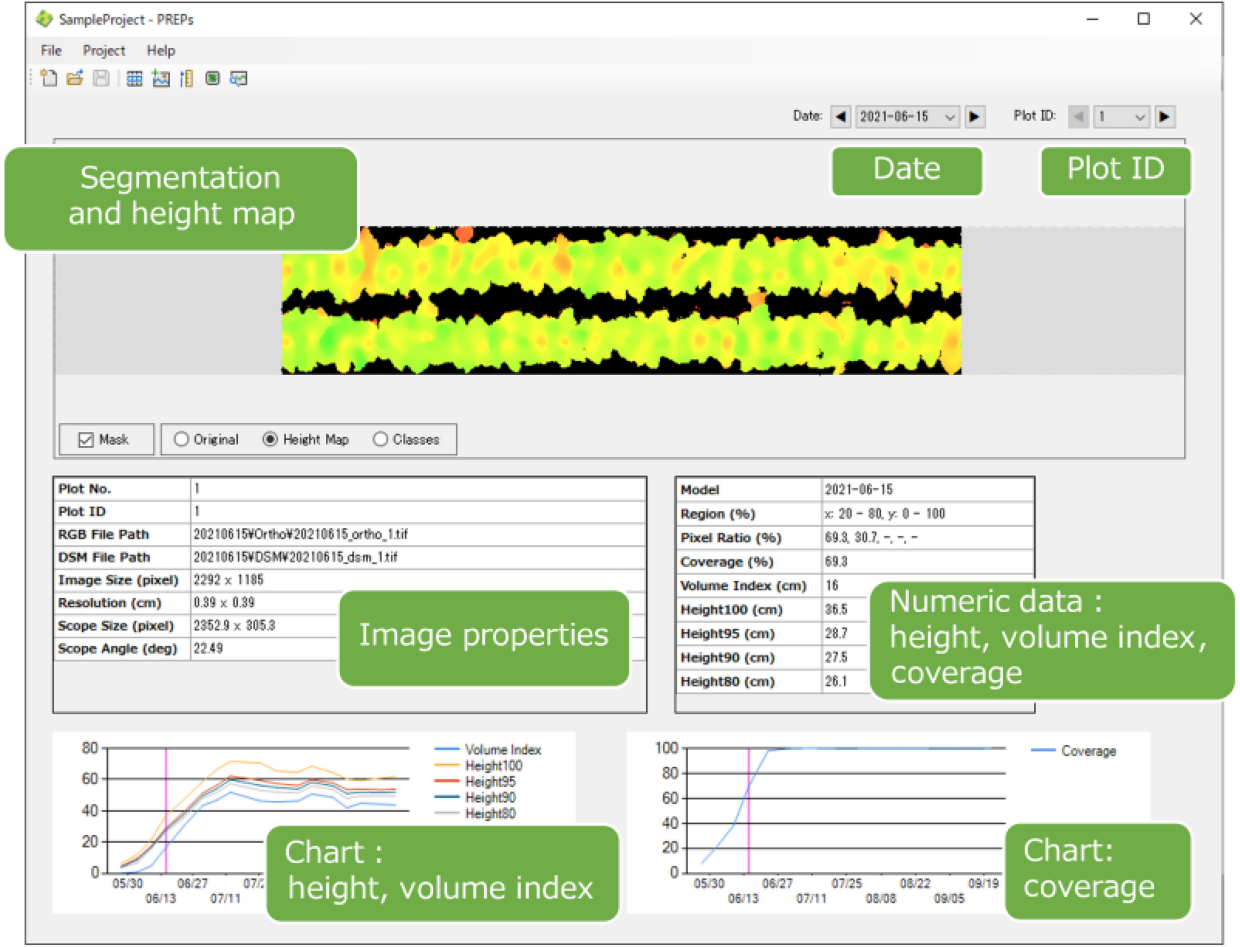
General overview of the PREPs software and its respective functions.

### Definition of microplots

In breeding research, experimental areas are often divided into microplots, and it is a common practice to perform data processing for each plot. The numerical size of microplots would vary depending on whether they are based on individual plants, clustered or in a treatment area. The geospatial information of the microplots was defined using Shapefile. A Shapefile is a vector data format that is interoperable among geographical information system (GIS) and enables mutual utilization. Although GIS software is typically employed for creating Shapefiles, a certain degree of training is required to become proficient in its operation. Conversely, utilizing PREPs allows for the creation of Shapefiles containing information about multiple microplots through straightforward actions, such as specifying image rotation (angle) and rectangular dimensions, followed by dragging rectangles within the image using the mouse. Once the Shapefile is created, the subsequent addition of images triggers automatic segmentation of the images into microplots.

### Calculation of base plane

In the DSM, elevation is recorded, making it possible to obtain crop heights by calculating the difference in height between the crop and bare ground. However, in images covered by crops, the bare ground directly underneath the crops was not visible, preventing the direct calculation of these differences. Therefore, it is necessary to obtain the ground elevation information in advance. Owing to the possibility of the field being inclined, the ground elevation within a single plot may not remain constant. Consequently, the ground is assumed to be a plane with a certain slope, referred to as the “base plane.” The only task that the user needs to perform to calculate the base plane is to indicate the area in the image where the bare ground appears as a rectangle. While preparing an image with bare ground is ideal, if unavailable, one can designate areas within the image where the ground is visible by drawing several rectangles, as illustrated in Fig. 2.

A large number of rectangles and a wider distribution of rectangles are expected to yield better results. Given a set of rectangles, the system calculates the least squares plane for a set of points within its range. From this plane, the system shifts the plane parallel to align with the point that has the furthest downward distance within the points, excluding the top 5% of the points by the vertical downward distance. The plane obtained through this process is the base plane. The exclusion of the top 5% of the points prevented the influence of locally anomalous values. The system also offers an alternative method for creating a base plane using thin lines rather than rectangles. For example, some crops are cultivated on raised beds. In such cases, it may be desirable to use the centerline of these rows, representing the peak height of the beds as the base plane. When specifying the elongated areas, if narrow rectangles are used, the limited number of samples along the shorter side may result in unexpected slopes in that direction. To avoid this, when designating elongated areas, it is recommended to use “line” option rather than rectangles. When using this option, the system automatically adjusts the base plane to ensure that the slope along the normal direction of the line is horizontal. The base plane is internally stored as a plane equation in the UTM coordinate system. Consequently, this plane serves as an absolute reference for all-time series data across different periods.

### Generating a learning model for cropcoverage estimation

**Fig. 3.**
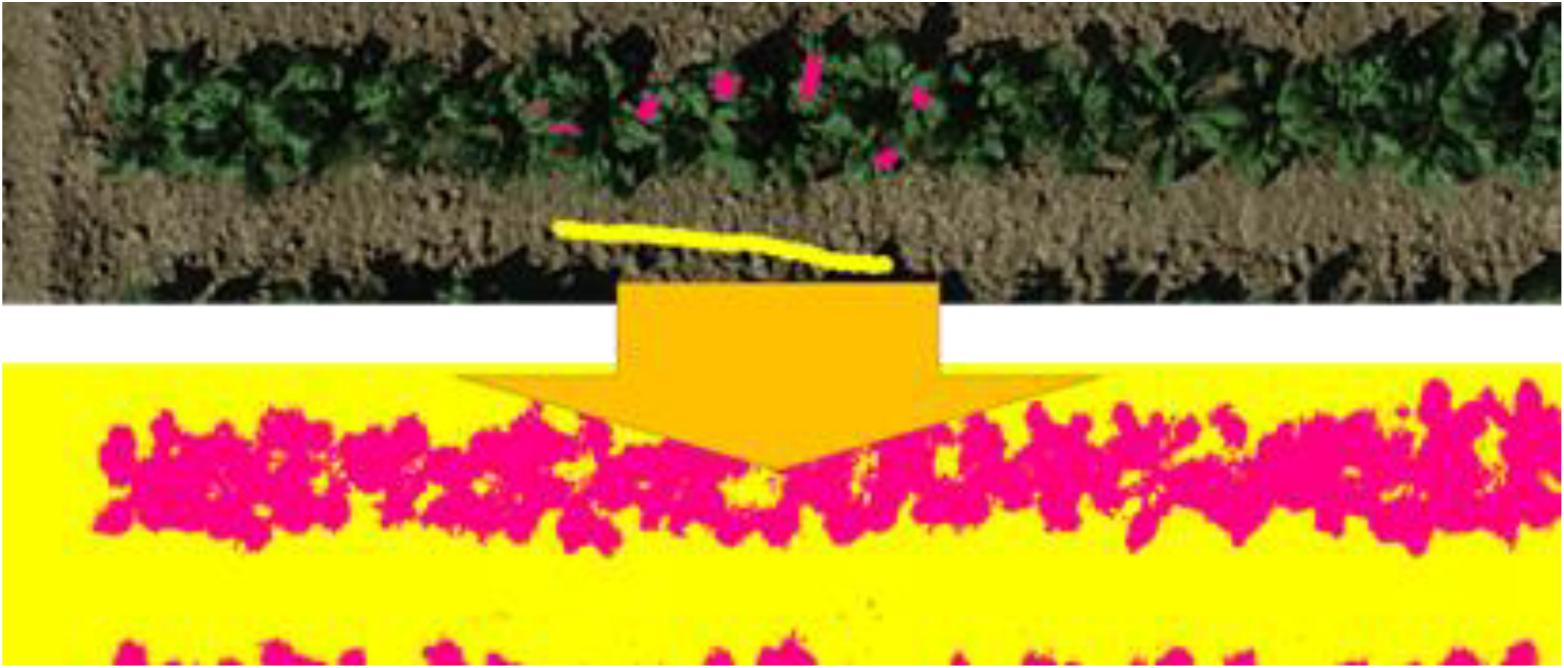
Segmentation of the crops from the soil by marking using a mouse where crops pixels (green) are segmented to pink color, while soil (brown) is segmented to yellow color.

Coverage, also known as the vegetation cover ratio, refers to the proportion of the projected area of the ground occupied by plants within a field. This is commonly used as an indicator of crop growth. The coverage is calculated using machine learning to determine whether each pixel within an image represents vegetation or something else. As a machine learning engine, the system employs the stochastic dual-coordinate ascent maximum entropy (SdcaMaximumEntropy) model included in Microsoft’s ML.NET. Users mark certain areas within an image using a mouse to indicate vegetation or nonvegetation. Machine learning was conducted based on the color information of the marked pixels. Similar to the method proposed by [1,14], in addition to RGB, HSV, and Lab, color spaces were also incorporated as part of the training data. The system allows a maximum of 5 classes to be defined, as shown in Fig. 3. They can be classified into 3 classes: petals, leaves, and others. However, because classification relies on color information, this method cannot be applied to objects that are indistinguishable based on color.

### Analysis of GeoTIFF files

After completing the 3 processes of defining the microplots, calculating the base plane, and generating the coverage model, as previously stated, the analysis of each GeoTIFF can be automated through batch processing. For each microplot within each GeoTIFF, the crop height, coverage, and volume index were estimated from the image data. Regarding the crop height, the height distribution across all pixels in the image was visually represented using color-coded visualization. Additionally, 4 metrics, namely, Height100, Height95, Height90, and Height80, were computed. For every pixel in the DSM, the vertical distance from the previously calculated base plane was measured to determine the crop height. Height100 represents the longest distance from the base plane among all the pixels. Height95 corresponds to the longest distance among the remaining pixels, after excluding the top 5% with the longest distance from the base plane. Similarly, Height90 and Height80 involve processing the remaining pixels after removing the top 10% and 20% of the longest distances from the base plane, respectively. Height100 indicates the tallest crop height within a plot. However, because of the potential influence of localized outlier values, it is recommended to refer to other metrics for assessing the overall crop height of the vegetation. The choice of metric to use should be made carefully, considering the intended use of the data and the characteristics of the crop. In relation to the vegetation coverage, all pixels in the image were classified and visually displayed using the learning model. The percentage occupied by each class was calculated simultaneously. Furthermore, vegetation coverage was automatically calculated by specifying which classes corresponded to the vegetation in advance. Moreover, nonvegetated pixels were used to mask the ground area during crop height visualization, while the vegetation pixels obtained from this training were used to identify the vegetation range during the subsequent volume index calculation.

The term “volume index” is defined as the average crop height of all areas, including the ground within the plot. Because point clouds do not account for blind spots during capture, an accurate assessment of crop volume is unattainable. Nevertheless, the cumulative value of the crop height across all pixels is believed to be highly correlated with the actual crop volume. In addition, the average height of all pixels, including those corresponding to the ground, reflected the vegetation quantity within the plot. Therefore, the volume index represented the amount of vegetation per unit area. Suitable units for the volume index are (cm^3^/cm^2^) or (cm). In the actual computation, the volume index was determined by summing the crop heights of all pixels corresponding to vegetation and then dividing this sum by the total number of pixels within the plot.

## Use Cases

### Use case 1: Monitoring the growth of sugar beet varieties using UAV

**Fig. 4.**
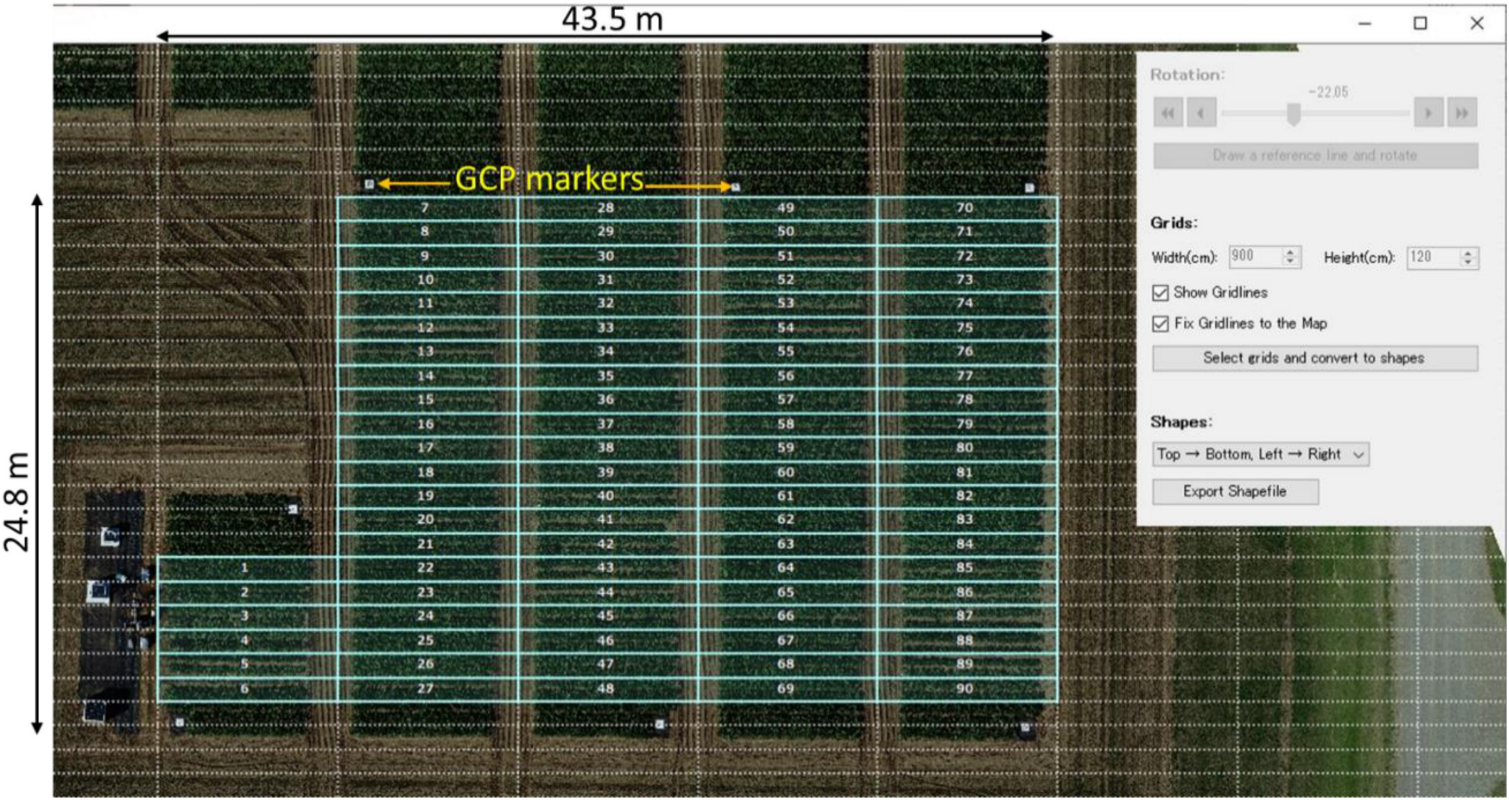
Experimental field where sugar beet varieties are grown, with numbers indicating the plots.

A 24.8 m x 43.5 m experimental field was utilized for growing the transplanted sugar beet varieties. The field consisted of both varieties and their F1s. Four replicates of the varieties and their F1 varieties (with each replicate having 20 plots consisting of 30 plants) were transplanted into the experimental field, as shown below. They were first sown in a greenhouse on 2021 March 11, after which they were transplanted to the field (Fig. 4) on 2021 April 22. A DJI Phantom 4 Pro (SZ DJI Technology Co. Ltd.) UAV fitted with an RGB camera was used for capturing RGB images in the field. Images were captured at a UAV height of 15 m with a capture interval of 2 s, a shutter speed of 1/1,000 and an ISO of 100. The 20-megapixel camera had a focal length of 24 mm. Images were captured at both nadir (90°) and oblique (45°) angles, with 7 GCPs evenly placed at the corners and center of the field. The GCPs were pre-measured using GPS-RTK with an accuracy of ±2 cm. The commercial SfM software Pix4D was used to generate 2 GeoTIFF files: orthomosaic RGB and DSM.

**Fig. 5.**
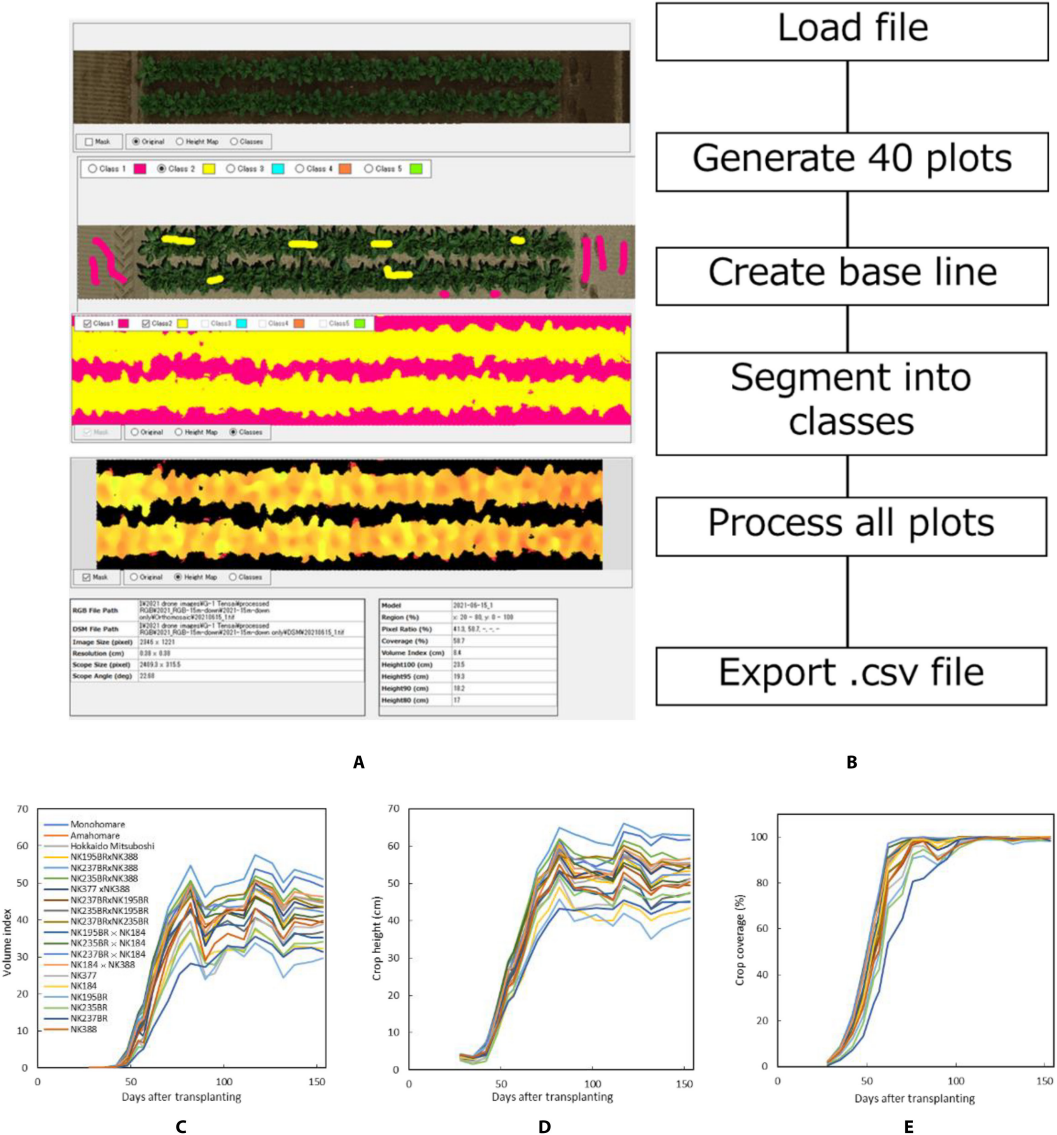
Using PREPs to process plots from sugar beet fields by (A) segmenting the crops from the soil and (B) the process flow diagram and time series data for (C) the volume index, (D) crop height, (E) crop coverage of all varieties.

Using the PREPs software, a new project was created, after which an orthomosaic file was loaded to define the boundaries for each sugar beet cultivar planted on a 2-row basis with a plot measuring 9 m × 1.2 m each. By setting the grid lines and converting them into a Shapefile, 40 plots were generated. Using the bare ground soil data, a base plane was calculated by drawing a rectangle in one of the plots; thus, minute unevenness of the ground could be ignored. A vegetation coverage model was generated using mouse markings in one of the plots containing both crops and bare soil in 2 classes: the green pixels representing the crops labeled as class 1 and the brown color of the soil labeled as class 2. Because of the time-series differences in lighting conditions, while capturing the images, a vegetation coverage model was created for each day, as shown in Fig. 5A. Because the plot area also includes bare ground soil on the sides where the tractor normally conducts operations, 20% of the side of the plot was cut off during processing to ensure accurate coverage and volume index estimation. The processing flow of the UAV-obtained DSM and orthophotos in the PREPs is shown in Fig. 5B.

The time-series data of the sugar beet varieties and their F1s are shown in Fig. 5C to E, which represent the changes in the crop volume index, height, and coverage, respectively. Clear differences were observed in the growth of these varieties and the F1s.

### Use case 2: Monitoring the growth of potato varieties using UAV

**Fig. 6.**
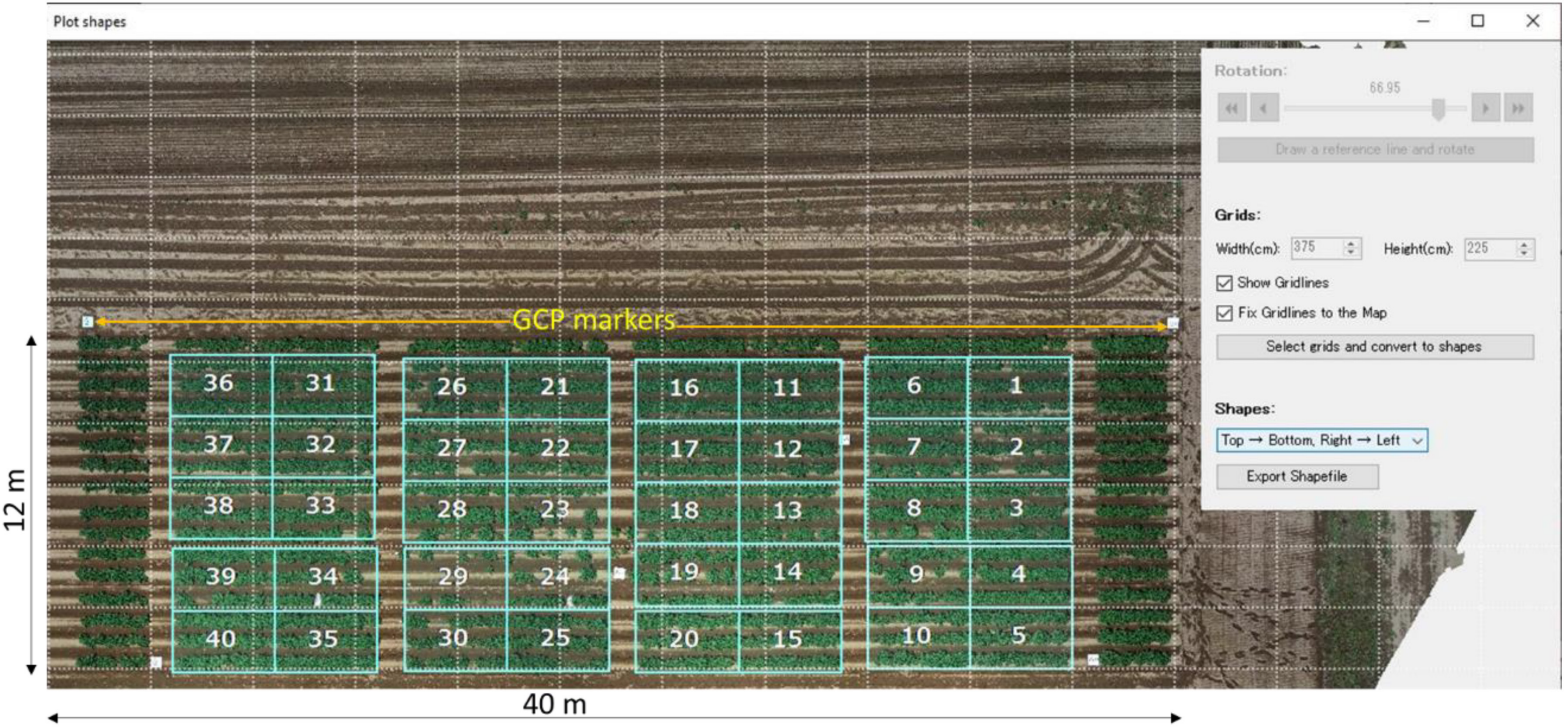
Experimental field showing both Japanese and European varieties represented by plot numbers.

The growth of 6 European and 3 Japanese varieties was monitored in an experimental field by using a UAV. The European varieties included Euroviva, Madison, Sorentina, Ivetta, Etana, and Georgina. The Japanese varieties of Toyoshiro, Konahime, and Irish Cobbler (locally known as Danshaku-imo) were used. Before planting, medium-sized potato seed tubers were cut into 2 pieces, after which they were planted at a height of 30 cm and an inter-row distance of 75 cm. Each variety was planted in 3 rows over an area of 3 m × 2.25 m as shown in Fig. 6.

In the potato experimental field, images were captured at a nadir (90°) with a front overlap of 80% and a side overlap of 75%, with the UAV flying at an altitude of 15 m above the ground surface. Four GCP markers were placed at the corner and 2 inside the plot for precise geocorrection of the maps. A DJI Phantom 4 was used to capture images in the field, and the camera settings were similar to those mentioned in case 1.

**Fig. 7.**
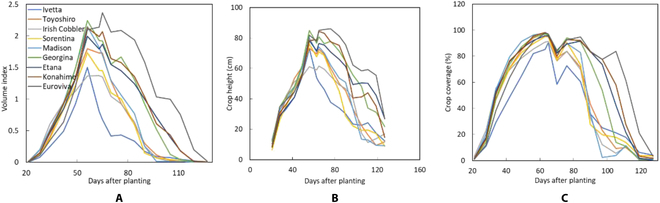
(A to C) Time series data of Japanese and European varieties showing changes in the volume index, crop height, and coverage.

The orthomosaic and DSM images were uploaded to the PREPs from which Shapefiles measuring 3 m × 2.25 m denoting the plot boundaries were created using the grid function. Potatoes are cultivated on ridges, which implies that even under bare ground conditions, the field already has undulations owing to ridges. Therefore, the base plane was calculated by specifying the central line of the ridge within the bare-ground images using a thin line, instead of the rectangle mentioned above. This made it possible to perform stable estimations of plant height without being affected by variations in ridge elevation. Crop coverage was generated using 2 classes: Class 1 represented the green pixels of the crops, and class 2 represented bare soil cover. All these were easily performed by labeling each day’s data with a mouse to create crop coverage models for analyzing the time-series data. The potato crop and soil were accurately segmented, and the time-series phenotypic traits were extracted, as shown in Fig. 7.

Varietal differences in volume index (Fig. 7A), crop height (Fig. 7B), and crop coverage (Fig. 7C) were observed. While European varieties of Euroviva and Georgina showed high values of volume index, crop height, and crop coverage, Japanese varieties of Irish Cobbler and Toyoshiro showed relatively lower values for these phenotypic traits throughout the growing season.

**Fig. 8.**
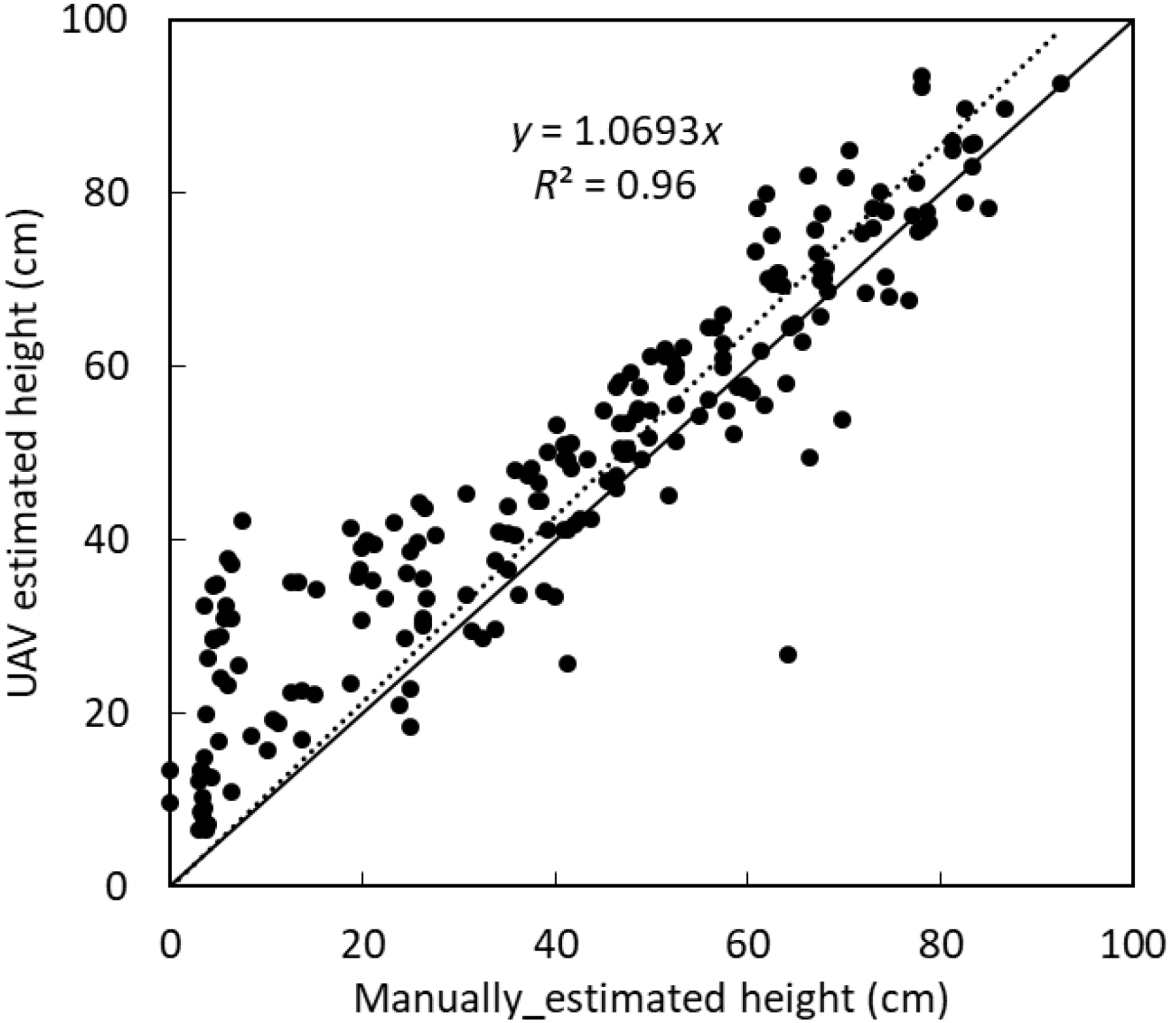
Comparison between UAV-estimated potato crop height and the manually estimated crop height.

A comparison was made between UAV-estimated crop height and the manually estimated crop height as shown in Fig. 8. The manual crop height was estimated weekly from each plot using a meter rule placed at the top of the ridge to measure the crop height close to the apex. From each plot, the height of 5 sample crops was measured and recorded, from which the average height of potatoes from each plot was estimated. It was found that a high correlation with a coefficient of determination of 0.96 was obtained between the 2 datasets.

### Use case 3: Monitoring the growth of potatoesusing multicamera array

**Fig. 9.**
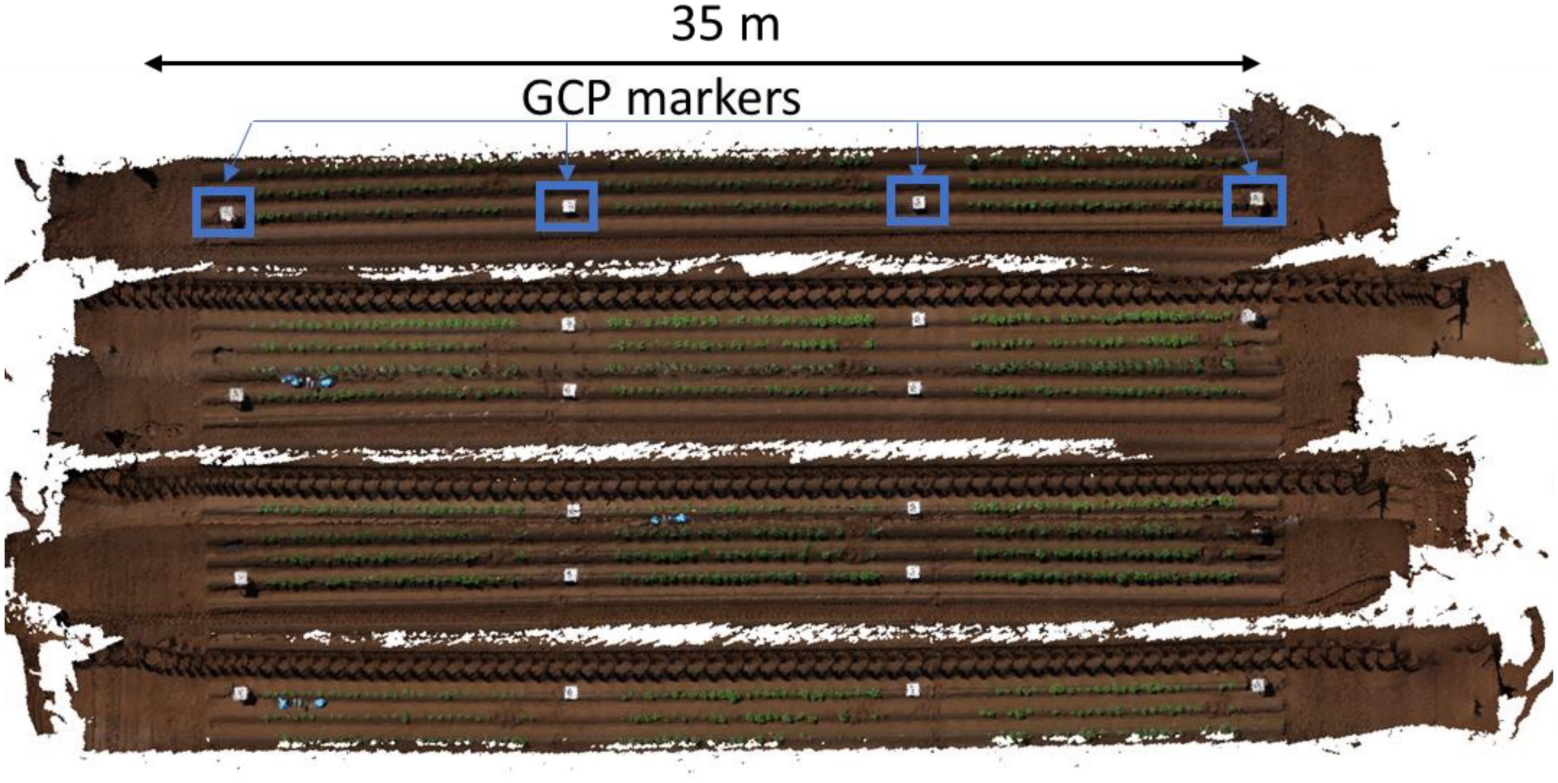
Orthomosaic maps produced by the multicamera system attached to a moving tractor.

A close-proximity multicamera array consisting of 6 cameras was used to obtain images from a potato experimental field. It consists of 6 RX0 II(DSC-RX0M2) cameras (Sony Corporation) mounted on a horizontal bar attached to the tractor and set 2.75 m above the ground surface. All cameras were set at a shutter speed of 1/1,000 and could take images at 1-s intervals with the ISO set at 100. The cameras were arranged in both nadir and oblique directions to ensure sufficient front overlap of the captured images. Furthermore, to ensure sufficient side overlap, the cameras were interspaced at 20-cm intervals. The growth of 2 Japanese varieties, Konahime and Touya, was monitored, and their phenotypic traits were estimated over time. Pre-cut medium-sized tubers were hand-planted at crop spacing and inter-row distances of 30 and 75 cm, respectively. The height of the potato crops was measured manually and compared with the height estimated using a multi-array camera system. The orthomosaic maps generated by the multicamera system attached to the tractor are shown in Fig. 9. The tractor moves along the potato ridges once each map is generated. Six maps were generated when the tractor moved along the potato ridges.

**Fig. 10.**
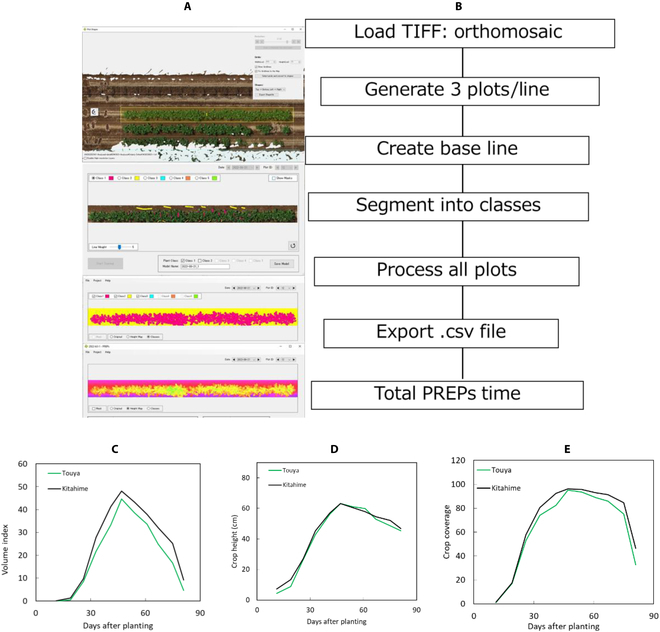
Using PREPs to process plots derived from multicamera-obtained data where (A) the potato crops are segmented from the soil and (B) the process flow from loading the orthomosaic images to generating phenotypic traits of (C) volume index, (D) crop height, and (E) crop coverage.

From the DSM and orthomosaic generated by the multicamera systems, 3 plots measuring 9 m × 0.75 m were created using the grid function from which Shapefiles were exported. The method for calculating the base plane is the same as that used in case 2. The crop coverage models were generated by marking the green pixels representing crops and the brown pixels representing the bare soil ground to segment the crops from the soil, as shown in Fig. 10A and B. All plots were processed from the time series data of crop height, volume index, and coverage file, as shown in Fig. 10C to E.

In both the volume index and crop coverage, Touya had a higher value throughout the growth period. However, there were no observable differences in crop height between the 2 varieties.

## Discussion

Using PREPs, the phenotypic traits of height, coverage, and volume index can be extracted from all use cases involving sugar beet and potato crops. The time taken to process the data from loading the files to creating microplots and finally analyzing the data for all the use cases was about 7 min.

Variations in the growth of sugar beet varieties and their F1s were observed when the crop height, volume index, and coverage were estimated. While crop height measurements can indicate the rate of growth of the crops, volume index shows the foliage density that is related to the amount of photosynthesis that occurs. Feng et al. [15] showed that light interception and utilization efficiency directly affect the canopy structure, thus affecting the photosynthetic activity of the phenotypes. Although higher volume index and foliage were observed in some varieties, such as NK235BR×NK388BR, this can also translate into increased yields because of the conversion of chlorophyll from the leaves to the sugar beet. Therefore, precise plant height and volume index measurements are ideal for monitoring the growth of sugar beet varieties.

European varieties, such as Euroviva, showed higher phenotypic traits of height, volume index, and coverage throughout the growth period. This is because they not only are late-maturing varieties but also have been reported to have high yields translated from high foliage volume and canopy coverage. A multicamera array could precisely extract the phenotypic traits of height, volume index, and coverage from the Touya and Kitahime potato varieties. The multicamera system, which was attached to a tractor, can monitor the growth of crops as the tractor moves along the potato field during crop operation activities, such as spraying, thus saving time for flying UAVs to capture images in the field. Furthermore, such high-resolution images are ideal for extracting extremely fine phenotypic traits, such as flower or bud information. While above-ground biomass has been used to predict yield in potatoes [16], the traits extracted from PREPs software such as volume index are expected to be utilized for yield prediction as more detailed canopy information can be extracted from volume estimation.

PREPs enables the extraction of crop traits of height, coverage, and volume. Using PREPs, breeders can selectively adjust the microplot sizes based on the layout of their phenotypic traits in outdoor fields, a feature that is unique to PREPs software. Furthermore, by using a mouse, simple machine learning segmentation can be done even with different shooting conditions by marking pixels related to either the crops or soils and creating training data, thus ensuring precise segmentation and precise training data by expert breeders for precise phenotyping. However, since the users have to create training datasets, further improvements would enable the automatic extraction of crop traits using pre-trained models. While processing data, a live graph displayed on PREPs interface enables the users to interactively understand their data trends. PREPs is advantageous since there is no need to learn about machine learning methods using Python, which takes time to learn and requires high-level skilled programming. Furthermore, since it is an open-source software, it overcomes the limitations of using currently commercial softwares that are expensive to buy and requires time to modify or learn in order to suite the users’ needs.

PREPs works on local environment of the computer. However, PREPs is currently only limited to 64-bit Windows OS platforms. Furthermore, before installation of PREPs software, it is necessary to install GDAL (Geospatial Data Abstraction Library) and set up the system environment variable in the computer properly. To enable the smooth installation, instructions for GDAL installation and configuration of computer environment have been attached in the PREPs software download link. Furthermore, PREPs version compatible with either GDAL version 3.6 or version 3.7 has been prepared for download and installation. Further work is required to test the accuracy of PREPs extracted trait data to demonstrate that the software can be effectively used in comprehensive breeding and agronomic studies.

## Conclusion

An easy-to-use Windows-based software, PREPs, was developed to analyze orthomosaic and DSM images. PREPs can process images not only obtained through UAV aerial photography but also generated by a multicamera system. PREPs is an open-source software that can be executed on any Windows computer as an executable program, allowing users to process their data without any programming skills. The creation of Shapefiles defining microplots, generation of training data for image segmentation, generation of base planes for plant height calculation, and other operations were all performed using simple mouse-centric actions. The time required for each operation was approximately 2 min per image set. The original images were automatically segmented on a microplot basis, and batch processing was employed for the automated analysis of these images, which required approximately 6 min. These functionalities eliminate the need for traditionally required tasks such as plot definition and elevation difference calculations using GIS, the utilization of machine learning models through programming languages, and the description of batch processes for repetitive tasks. Advanced skills conventionally essential for these tasks are unnecessary. As a proof of concept, PREPs has demonstrated the ability to efficiently acquire plant height, coverage, and volume index throughout the growth period. It can function accurately in crops with distinct characteristics such as sugar beets and potatoes. Furthermore, it exhibited the correct functionality across images with significantly different resolutions, including UAV and multicamera system images. Utilizing PREPs is particularly advantageous because it automates the generation of crop properties, eliminating the need for intricate programming and the difficulty in setting up specific computer environments for each software.

## Data Availability

PREPs software is available for free under MIT License, and the instructions for installations together with sample images are also attached at: http://cse.naro.affrc.go.jp/aitoh/PREPs/?en.
